# A Case Report of Atypical Hemolytic Uremic Syndrome in a Two-Month-Old Infant With a Negative Reported Genetic Profile and Five-Year Follow-Up on Eculizumab

**DOI:** 10.7759/cureus.10392

**Published:** 2020-09-11

**Authors:** Siddharth Shah, Laith Sweis

**Affiliations:** 1 Pediatric Nephrology, Norton Children's and University of Louisville, Louisville, USA; 2 Pediatrics, Norton Children's and University of Louisville, Louisville, USA

**Keywords:** ahus, eculizumab, cardiomyopathy, dilated cardiomyopathy, chronic kidney disease

## Abstract

Atypical hemolytic uremic syndrome (aHUS) is a rare but life-threatening pediatric disease caused by uncontrolled activation of the alternative complement pathway related to genetic mutations and carries a worse prognosis. In the last decade, a monoclonal antibody against complement C5, eculizumab, has dramatically improved the disease outcomes. The complement mutations in aHUS are detected only in 60%-70% of cases in previous studies. We report a severe presentation of aHUS diagnosed in a two-month-old child who presented with seizures, renal failure with anuria, and microangiopathic hemolytic anemia and required peritoneal dialysis soon after admission. The patient was clinically diagnosed having aHUS and was started on eculizumab on day 4 of hospital admission. The genetic study for major known complement mutations causing aHUS was reported negative. He had a major episode of disease relapse associated with seizures four weeks after eculizumab therapy and required prolonged peritoneal dialysis over more than two months at the time of initial admission. He developed dilated cardiomyopathy and oro-motor dysfunction as complications of aHUS. At five-year follow-up, the patient has stage 3 chronic kidney disease (CKD), proteinuria, hypertension, and required G-tube for feeds. This report discussed the long-term outcome of an infant diagnosed with aHUS and tested negative for common complement mutations on eculizumab therapy. More research is needed to identify novel genes and antibodies contributing to aHUS. While the eculizumab is expensive, and the duration of treatment is not definite, the clinical severity of the disease, relapses, and presence of long-term renal complications are essential factors to decide treatment continuation.

## Introduction

Hemolytic uremic syndrome (HUS) presents with microangiopathic hemolytic anemia, thrombocytopenia, and acute renal failure and is one of the prominent causes of acute kidney injury in children [1]. Typical HUS, also known as Shiga toxin-producing Escherichia coli‑associated HUS (STEC-HUS), is a common cause of HUS in pediatric patients and has a <5% mortality with early supportive treatment [1]. By contrast, atypical HUS (aHUS) in pediatric patients is a rare disorder with a reported incidence of 0.10-0.11 cases per million, constitute about 10% of all HUS cases, is primarily related to uncontrolled complement activation caused by genetic factors or auto-antibodies, and has a poor prognosis [2-5]. Previously, aHUS was managed with supportive care, dialysis, plasma exchange, and infusions; however, 33%-40% of patients had end-stage kidney disease or death as the initial disease manifestation, and over 50% progressed to end-stage renal disease (ESRD) [4-5].

Over the last decade, eculizumab, a monoclonal antibody that binds to complement C5 and attenuates complement hyperactivity, has positively changed the outcome of children with aHUS and is now the current standard of care [4-5]. Complement gene mutations were detected only in approximately 60%-70% of patients with aHUS in previous studies [3, 6].

## Case presentation

A previously healthy two-month-old male (weight: 5.74 kg), born at full term without complications, presented to the ER with a four-day history of nonbloody, nonbilious emesis, and nonbloody loose stools. On presentation to the ER, he was lethargic and had an episode of seizure. The family history was negative for kidney disease.

The initial laboratory studies were significant for severe anemia, with hemoglobin (Hgb) of 5.4 g/dL, and for thrombocytopenia, with a platelet count of 37 × 103/µL. The initial white blood cell (WBC) count was 16.1 × 103/µL, and Hgb was low, at 5.4 g/dL. Peripheral smears showed the presence of schistocytes. Serum chemistry showed sodium at 126 meq/L, potassium at 5.7 meq/L, chloride at 94 meq/L, bicarbonate at 17 meq/L, blood urea nitrogen (BUN) at 87 mg/dL, serum creatinine at 3.9 mg/dL, aspartate aminotransferase (AST) at 163 U/L, and alanine aminotransferase (ALT) at 134 U/L. His serum lactate dehydrogenase (LDH) level was >6450 U/L (reference range: 313-618 U/L). His stool culture was negative for Shiga toxin and entero-hemorrhagic E. coli 0157:H7. The complement C3 level was low, at 72 mg/dL (reference range: 80-181 mg/dL). The complement C4 was 13 mg/dL (reference range: 12-52 mg/dL). The workups for possible infectious and bone marrow diseases were largely unremarkable (see Table 1).

**Table 1:** Infectious disease, genetic, and malignancy work-up results during hospitalization. CSF, cerebrospinal fluid; PCR, polymerase chain reaction

Test	Result
CSF culture	Negative
Blood culture	Negative
Urine culture	Negative
Stool culture (viral/bacterial)	Negative
Herpes simplex virus PCR, CSF	Negative
Cytomegalovirus PCR, urine	Negative
Shiga Toxin	Negative
Microarray result of whole genome	Normal male
Bone marrow	No abnormal cell lines or findings consistent with malignancy were noted

Diagnostic assessment

A clinical diagnosis of thrombotic microangiopathy (TMA) was made based on the patient’s renal failure, neurological symptoms, and gastrointestinal symptoms, as well as on evidence of microangiopathic hemolysis (as suggested by the presence of schistocytes on the peripheral smear), hemolytic anemia with high lactate dehydrogenase (LDH) counts, and thrombocytopenia. We performed antibody, complement, and genetic evaluations for further evaluation of TMA (see Table 2). The ADAMTS13 activity was only slightly low, at 53% (normal >61%), and was not suggestive of thrombotic thrombocytopenic purpura (TTP). No causes of secondary HUS related to infectious diseases, bone marrow diseases, or immune system disorders such as hemophagocytic lymphohistiocytosis (HLH) were identified.

Typical HUS rarely presents in infants less than six months of age, so the suspicion of aHUS was high [7]. The stool study also did not detect Shiga toxin. Diarrhea can occur in aHUS cases [4]; therefore, our diagnostic suspicion was not altered by the presence of diarrhea at presentation. The genetic studies did not identify the presence of common complement mutations likely to cause aHUS in our patient (see Table 2).

**Table 2:** Thrombotic microangiopathy work-up. The reference range is provided in brackets.

Test	Result
ADAMTS13 activity	53% (>61%)
C3 complement	72 mg/dL (81–181 mg/dL)
C4 complement	13 mg/dL (12–52 mg/dL)
Plasma homocysteine	10.8 µmol/L (6.6–14.8 µmol/L)
Plasma methylmalonic acid	0.4 µmol/L (0–0.4 µmol/L)
Complement factor H	296 mcg/mL (160–412 mcg/mL)
Membrane cofactor protein, CD46 expression	Polymorphonuclear leukocyte ratio 0.86 (0.73–1.31)
Factor H auto-antibody	<= 22 unit/mL (<=22 unit/mL)
Factor I auto-antibody	3.1 mg/dL (2.4–4.3)
Atypical HUS genetic panel [CFH, MCP (CD46), CFI, C3, CFB, CFHR1, CFHR3, CFHR4, CFHR5, thrombomodulin (THBD), plasminogen (PLG) and DGKE]	No disease-associated mutations identified
Hemophagocytic lymphohistiocytosis genetic panel	No mutation identified
Complement Bb serum	10.693 (2.749–116.333)
Peripheral smear	Marked normocytic, hypochromic anemia with schistocytes

The complement C3 level was low, but we did not check the C5b-9 (membrane attack complex; MAC) level as part of the initial TMA evaluation. We tested the C5b-9 level later to monitor his response to eculizumab and his relapse state.

Therapeutic assessment

A peritoneal dialysis catheter was placed, and peritoneal dialysis had to be initiated within 24 hours of hospital admission for acute renal failure with anuria and volume overload. Within the first 24 hours of hospital admission, the patient had another episode of seizure and required intubation for respiratory support. He was also administered a plasma infusion (PI), given the suspected presence of aHUS. While still awaiting the genetic study results at four days after hospital admission, we started the patient on eculizumab treatment because of the suspected aHUS. The persistence of neurological symptoms and the ongoing requirement of respiratory support led to this decision. The patient was also administered the meningitis vaccine for meningitis prevention and was started on penicillin prophylaxis before initiation of the eculizumab therapy.

The patient showed evidence of hematological remission, with improvement in platelet count to 163 × 103/µL and an LDH level trending down to 450 U/L, at two weeks after the initiation of eculizumab. His respiratory status also improved, and he no longer required respiratory support. He continued to require peritoneal dialysis because of persistent oligo-anuria.

However, four weeks after the initiation of eculizumab, and while maintained on weekly eculizumab infusions, the patient had another episode of prolonged seizure activity. An infectious workup, including cerebrospinal fluid (CSF) culture, was repeated and was negative. A brain MRI was unremarkable. The platelet counts started to drop again to a low of 36 × 103/µL, and the LDH level rose to a peak of 5058 U/L. More remarkably, the WBC count peaked at 88 ×103/µL. We diagnosed this episode as aHUS relapse that happened after two weeks of hematological remission. The C5b-9 level, checked at the time of the relapse, was adequately suppressed at 132 ng/mL (reference < 244 ng/mL), and the total complement (CH50) was low, at six units (reference range: 60-144 units).

Eculizumab was given as per the induction schedule weekly for six doses and was then changed to a maintenance schedule dosing every three weeks. The rationale for aggressive eculizumab therapy early in the course of the disease was related to the patient’s disease severity and the relapse episode.

Six weeks after the initiation of eculizumab, and after the relapse episode, the platelet counts again improved to >150 × 103 /µL and remained in the normal range without further episodes of relapse during the admission.

Follow-up and outcomes

Sixty days after his hospitalization, the patient developed left ventricular dilated cardiomyopathy with a left ventricular ejection fraction of 44% that was not previously noted on prior echocardiograms. He developed decompensated heart failure that required treatment with milrinone until his condition stabilized. His C5b-9 levels remained adequately suppressed while on eculizumab therapy, and the cardiac ejection fraction continued to improve on subsequent echocardiogram studies. His renal recovery was significantly delayed, even after hematological recovery after the relapse episode.

After 70 days of eculizumab therapy, peritoneal dialysis was discontinued, and the peritoneal dialysis catheter was removed, as the renal clearance function and urine output were improved. The serum creatinine reached a baseline of 0.6-0.7 mg/dL after discontinuation of dialysis. The patient was subsequently maintained on anti-hypertensives and electrolyte replacement therapy. In total, this patient remained hospitalized for 97 days following his initial hospital admission. The total complement CH50 remained adequately suppressed at less than 10% of the normal range, and the C5b-9 level remained suppressed while on eculizumab therapy. 

Five-year follow-up

The patient had brief episodes of hematological relapse in June 2019 and February 2020, when the LDH count was 907 and 1144 IU/L, respectively, and these episodes were associated with low-grade fever and viral infection. The platelet counts remained at more than 150 × 103/µL during this time, as the eculizumab therapy continued. The C5b-9 level remained suppressed during these episodes. The serum creatinine and urine output remained stable, and no neurological symptoms occurred. His renal function stabilized with eculizumab therapy, but the patient developed complications from his course of aHUS and his prolonged requirement for dialysis. The serum creatinine checked five years after the disease onset was 0.9 mg/dL.

The patient currently has stage III chronic kidney disease (CKD) with an estimated glomerular filtration rate (eGFR) of 50 mL/min/1.73 m2 measured through the revised Schwartz equation. He has proteinuria, with a random urine protein and creatinine ratio ranging between 1 and 1.5 (reference range less than 0.2). Lisinopril is being used to treat proteinuria and hypertension. Even with proteinuria, his CKD is only slowly progressive, and his eGFR has been maintained in a range of 50-60 mL/min/1.73 m2 while on eculizumab treatment for five years. He is diagnosed with a swallowing disorder as a sequela to aHUS and is dependent on a G-tube for feeds. He continues to be maintained on antiepileptic drug therapy. No adverse events, such as meningitis, were noted during eculizumab therapy. Although the studies on when eculizumab therapy can be safely stopped are lacking, we plan to continue this therapy due to concerns that another significant episode of relapse can lead to a substantial progression of the patient’s CKD.

## Discussion

Atypical HUS can have devastating outcomes in children [4-5]. Before the availability of complement-regulating monoclonal antibodies as a potential treatment for this disease, the mortality and ESRD rates from this disease were high. For example, one study reported that if oliguria lasted for more than 14 days or if anuria lasted for more than seven days, the chances of renal recovery from aHUS was only 13%. Most children had outcomes of severe CKD, end-stage kidney disease, and death [8].

Uncontrolled activation of the alternative pathway (AP) of complements leading to microangiopathic hemolytic anemia is central to the pathogenesis of aHUS [2, 5, 9]. The increased production of end products of the alternative complement pathway, like C5b-9, leads to endothelial damage and subsequent microvascular thrombosis in organs such as the kidneys, heart, and brain, leading to complications such as renal failure, seizures, encephalopathy, and cardiomyopathy [2, 5, 9].

Eculizumab works as a monoclonal antibody against C5, a key mediator of alternative complement pathway activation, and inhibits its conversion to the proinflammatory molecule ‘C5a.’ Eculizumab also decreases the terminal complement activation and formation of C5b-9, thereby preventing endothelial damage to the blood vessels in aHUS [5, 9-10]. Eculizumab has been proven as a safe and effective treatment for aHUS in children and has dramatically improved the outcomes and complications of this disease [10-11].

In this report, we discuss a severe presentation of aHUS clinically diagnosed in a two-month old infant. A suspicion of aHUS should be raised if the presentation of TMA occurs in an infant less than six months of age [7]. The clinical diagnosis of aHUS is challenging in small infants, but a delay in treatment can cause severe complications and death [12]. We started plasma infusion (PI) within 24 hours of diagnosis of TMA in our patient. However, literature reviews on aHUS have shown that PI therapy is not adequate for aHUS and that it increases the risk of volume overload and progressive renal impairment [5, 7]. Moreover, PI has a limited role in children with complement factor H mutations, but it is not helpful in patients with variations in the MCP (CD46) or CFI genes [3, 7]. One study has shown that rapid clinical diagnosis and early administration of eculizumab were critical for improving renal outcomes in patients with aHUS [13]. In our patient, we started eculizumab on the fourth day of hospital admission. However, he still had a significant episode of relapse even when on the induction eculizumab schedule, further suggesting the severity of his aHUS presentation.

Our patient had negative results for the genetic studies for typically reported aHUS mutations. To date, reports are not widely available on the long-term outcomes in children who test negative for prevalent complement mutations in aHUS and are maintained on eculizumab therapy. One study conducted before the emergence of eculizumab as a potential therapy reported that the consequences of ESRD or death were not significantly different in patients with and without detected complement mutations in aHUS [3]. The negative genetic findings in aHUS, more than anything, may reflect the present diagnostic limitations in detecting all existing complement pathway mutations. More research is needed in this area [14], and our case report also highlights a similar need.

Cardiovascular complications, such as cardiomyopathy, have been previously reported in 10% of patients with aHUS [15]. One case report previously discussed improvement in cardiac function in a four-year-old child with dilated cardiomyopathy as a sequela to aHUS following eculizumab therapy [16]. Our patient was also diagnosed with dilated cardiomyopathy as a sequela to aHUS, and we report a similar improvement in cardiac function during eculizumab treatment. Our patient also has a swallowing disorder secondary to oro-motor dysfunction as a sequela to aHUS, although his brain MRI did not reveal any neurological damage. This extra-renal manifestation of aHUS has not been reported previously, to our knowledge. He is still maintained on a G-tube for feeds, whereas his other gross developmental milestones are now appropriate for his age.

Another peculiar finding of our patient was a severe presentation of renal failure and a prolonged requirement for peritoneal dialysis over a period of more than two months during the initial hospital admission. Our patient had sustained a second episode of major aHUS disease relapse after four weeks of starting eculizumab, and he likely suffered another renal insult that resulted in his prolonged requirement for dialysis. Our patient currently has stage 3 CKD, proteinuria, and hypertension as sequelae to his aHUS-induced renal injury. A previous study discussed improvement in renal outcomes, including CKD and dialysis, in aHUS while on maintenance eculizumab therapy [11]. Our patient was able to get off dialysis during the initial hospital admission, and he has had a relatively stable progression of CKD (in the range of stage 3 CKD) for the past five years while on maintenance eculizumab therapy.

Attempts to guide the duration and dosing of eculizumab therapy still require much research in patients with aHUS. One study has suggested the use of MAC or C5b-9 as a marker to monitor complement inhibition [17]. A consensus guideline has discussed keeping the CH50 level at less than 10% of the normal range when deciding to change the dosing schedule [18]. Another report talked about maintaining the global classical complement pathway activity at less than 30% [19]. Elsewhere, the eculizumab trough level is mentioned as a better way to monitor disease activity and remission. An eculizumab drug level near 100 μg/mL was associated with markedly decreased CH50 activity [18]. Currently, we periodically measure C5b-9, C3a, and C5a levels to guide AP complement suppression. A report has suggested that while the cost can be as high as $700,000 per patient per year of treatment associated with aHUS, the estimated relapse rate is 30% following discontinuation of eculizumab [20]. The relapse rate is expected to be even higher in pediatric patients, according to the same report [20].

In the absence of an identified genetic mutation, the risk of relapse in our patient is unknown. A consensus guideline has suggested not withdrawing eculizumab in children with a severe presentation of aHUS and when kidney function has not fully recovered [18]. Based on the severity of aHUS in our patient, his episode of relapse, the presence of stage 3 CKD, and his brief rise in LDH count with recent viral infections, our plan is to continue eculizumab for maintenance at this time. We are highlighting the following teaching points from this case report (see Table 3).

**Table 3:** Teaching points. aHUS, atypical hemolytic uremic syndrome; TMA, thrombotic microangiopathy

No.	Teaching points
1	The clinical diagnosis of aHUS is challenging in small infants with diarrhea as a presenting symptom, and the treatment should not be delayed.
2	The suspicion of aHUS should be high if TMA presents in small infants.
3	aHUS relapse may present early in treatment course even while on eculizumab treatment, and clinicians should be vigilant to monitor it.
4.	There is a paucity of literature on long-term outcomes in children with aHUS and a negative genetic complement mutation profile. The decision to continue eculizumab in these patients may be based on disease severity, relapse, and the presence of renal impairment.
5	Standard monitoring guidelines need to be established to assess the response of eculizumab in children.
6	Oro-motor dysfunction and long-term G-tube dependence may be an extrarenal manifestation of aHUS not previously reported.

One limitation of our case report was that we did not test C5b-9, C3a, or C5a levels at admission; this would have provided more substantial evidence for alternative complement pathway activation. We also did not check the periodic eculizumab levels in our patient, but this testing was not substantiated or widely available at the time of the initial presentation of this patient.

## Conclusions

We report a severe presentation of aHUS with a major relapse in which eculizumab helped improve cardiovascular outcomes and aided in partial renal recovery during a five-year follow-up. More research is needed to identify additional complement mutations that might contribute to aHUS and to establish standard monitoring guidelines for eculizumab therapy in pediatric patients. The safe duration of eculizumab use is unknown; therefore, the clinical severity of the disease, any relapse, and the presence of renal impairment are essential factors when deciding on treatment continuation.
